# Alcohol use disorder and alcohol-related mortality after metabolic bariatric surgery: prospective controlled cohort study

**DOI:** 10.1093/bjs/znaf211

**Published:** 2025-10-09

**Authors:** Kajsa Sjöholm, Markku Peltonen, Peter Jacobson, Johanna C Andersson-Assarsson, Sofie Ahlin, Lucas Adméus, Ida Arnetorp, My Engström, Magdalena Taube, Lena M S Carlsson, Per-Arne Svensson

**Affiliations:** Department of Molecular and Clinical Medicine, Institute of Medicine, Sahlgrenska Academy at University of Gothenburg, Gothenburg, Sweden; Finnish Institute for Health and Welfare, Helsinki, Finland; Department of Molecular and Clinical Medicine, Institute of Medicine, Sahlgrenska Academy at University of Gothenburg, Gothenburg, Sweden; Department of Molecular and Clinical Medicine, Institute of Medicine, Sahlgrenska Academy at University of Gothenburg, Gothenburg, Sweden; Department of Molecular and Clinical Medicine, Institute of Medicine, Sahlgrenska Academy at University of Gothenburg, Gothenburg, Sweden; Department of Clinical Physiology, Region Västra Götaland, NU Hospital Group, Trollhättan, Sweden; Department of Molecular and Clinical Medicine, Institute of Medicine, Sahlgrenska Academy at University of Gothenburg, Gothenburg, Sweden; Department of Molecular and Clinical Medicine, Institute of Medicine, Sahlgrenska Academy at University of Gothenburg, Gothenburg, Sweden; Department of Surgery, Region Västra Götaland, Sahlgrenska University Hospital, Gothenburg, Sweden; Institute of Health and Care Sciences, Sahlgrenska Academy at University of Gothenburg, Gothenburg, Sweden; Department of Molecular and Clinical Medicine, Institute of Medicine, Sahlgrenska Academy at University of Gothenburg, Gothenburg, Sweden; Department of Molecular and Clinical Medicine, Institute of Medicine, Sahlgrenska Academy at University of Gothenburg, Gothenburg, Sweden; Department of Molecular and Clinical Medicine, Institute of Medicine, Sahlgrenska Academy at University of Gothenburg, Gothenburg, Sweden; Institute of Health and Care Sciences, Sahlgrenska Academy at University of Gothenburg, Gothenburg, Sweden

## Abstract

**Background:**

A body of evidence supports a link between metabolic bariatric surgery (MBS) and alcohol use disorder (AUD), while the possible contribution to alcohol-related mortality remains unclear. The aim of this study was to examine the association between MBS and the risk of AUD and alcohol-related mortality over up to 35 years.

**Methods:**

The Swedish Obese Subjects (SOS) study enrolled 2007 participants with severe obesity who underwent MBS and 2040 matched controls (median follow-up 25.2 years). Patients in the surgery group underwent gastric bypass (GBP; 266 patients), gastric banding (376 patients), or vertical banded gastroplasty (VBG; 1365 patients). The matched controls received the customary treatment for severe obesity at their primary healthcare centres. Data on AUD diagnoses and alcohol-related mortality were captured from the Swedish National Patient Register and the Swedish Cause of Death Register respectively.

**Results:**

During long-term follow-up, a significant difference in the incidence of AUD was found across surgery groups (log rank *P* < 0.001). Patients who underwent GBP exhibited the highest AUD risk (adjusted HR (HRadj) 5.07 (95% c.i. 3.11 to 8.25); *P* < 0.001), followed by patients who underwent VBG (HRadj 2.28 (95% c.i. 1.56 to 3.34); *P* < 0.001) and patients who underwent gastric banding (HRadj 2.34 (95% c.i. 1.37 to 4.01); *P* = 0.002), compared with usual obesity care. Alcohol-related mortality was significantly elevated after GBP (adjusted sub-HR (sub-HRadj) 6.18 (95% c.i. 2.48 to 15.40); *P* < 0.001) and VBG (sub-HRadj 3.56 (95% c.i. 1.79 to 7.08); *P* < 0.001) compared with usual obesity care. Mortality after gastric banding was also elevated, but did not reach statistical significance (sub-HRadj 2.52 (95% c.i. 0.89 to 7.15); *P* = 0.082).

**Conclusion:**

Effective management of alcohol-related complications in MBS patients requires preoperative risk assessment, postoperative monitoring, and access to targeted interventions for AUD.

## Teaser

This study examined the risk of alcohol-related mortality after bariatric surgery in patients from the Swedish Obese Subjects (SOS) study. The mortality risk was significantly higher in the gastric bypass and VBG groups compared with the control group.

## Introduction

In response to the global obesity epidemic, many patients with obesity have been treated with metabolic bariatric surgery (MBS). Such procedures lead to long-term sustained weight loss^1^ and are associated with a reduced risk of various obesity-related co-morbidities^2^, as well as an increase in life expectancy^1^. However, MBS is also associated with several short- and long-term side effects^2,3^.

Increased sensitivity to alcohol due to an altered alcohol metabolism has been observed, especially after gastric bypass (GBP) surgery^4–6^. Subsequently, several studies have suggested an association between MBS, especially GBP, and harmful alcohol use^7–10^. In 2016, the American Society for Metabolic and Bariatric Surgery issued a position statement highlighting an increased risk of developing alcohol use disorder (AUD) after GBP^11^. However, the existing body of literature on this topic remains inconsistent. Generally, long-term follow-up studies observe an elevated risk of alcohol-related problems^3,9,10,12–19^, whereas shorter-term follow-up studies sometimes observe no increased risk^20^ or even indicate reduced alcohol consumption and fewer alcohol-related problems^21–24^. Meta-analyses and systematic reviews further underscore this inconsistency, suggesting that long-term follow-up is needed to detect alcohol-related problems after MBS^25–27^, emphasizing the need for additional high-quality long-term research studies on this topic. Furthermore, data on the risk of alcohol-related mortality after MBS remain limited.

The Swedish Obese Subjects (SOS) study previously demonstrated an association between MBS and an elevated risk of both self-reported harmful alcohol consumption and alcohol problems, as well as register-based diagnoses of AUD^10^. More recently, findings suggest that post-surgery addiction issues may extend beyond alcohol to include other addictive substances^28^. The aim of this study was to investigate the long-term association between MBS and diagnosed AUD over a 35-year interval, as well as its potential impact on alcohol-related mortality.

## Methods

### Study design and participants

**Fig. 1:**
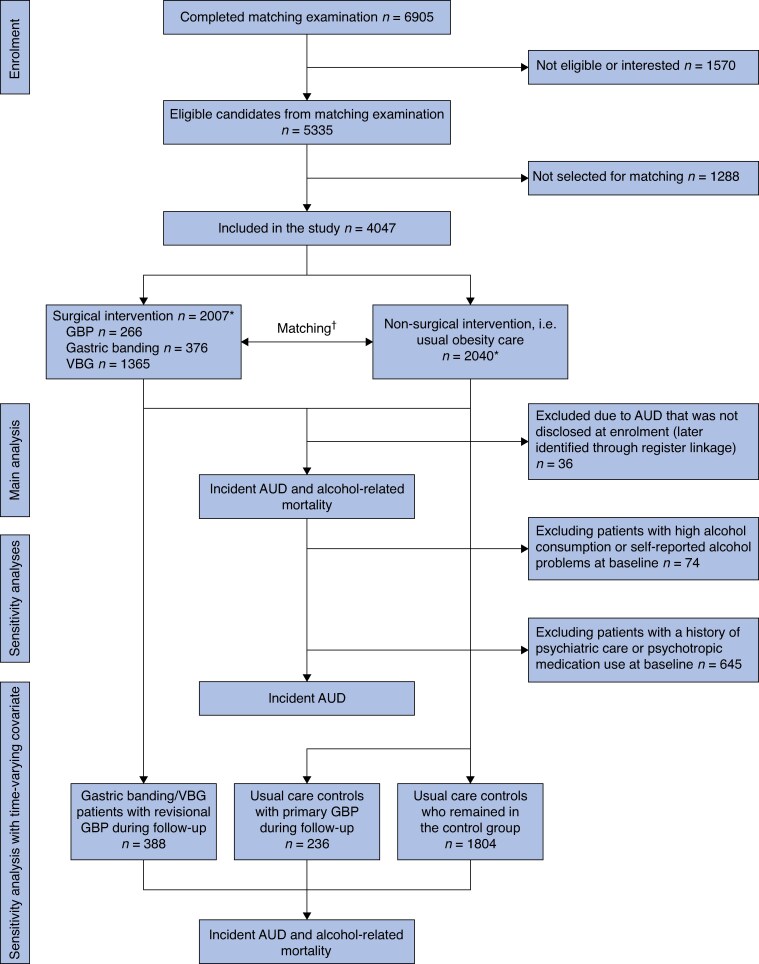
Flow chart describing inclusion and matching of participants in the SOS study and individuals available for analysis Originally, 2010 participants were recruited to the surgery group and 2037 individuals were recruited to the control group. However, 3 participants did not undergo the planned surgery, resulting in 2007 participants in the surgery group and 2040 participants in the control group, who were included in the per-protocol* analysis. †Groups matched by sex, age, weight, height, waist and hip circumferences, systolic blood pressure, serum cholesterol and triglyceride levels, smoking status, diabetes, menopausal status, four psychosocial variables with known associations with risk of death, and two personality traits related to treatment preferences. SOS, Swedish Obese Subjects; GBP, gastric bypass; VBG, vertical banded gastroplasty; AUD, alcohol use disorder.

The SOS study, conducted at 25 surgical departments and 480 primary healthcare centres in Sweden, has previously been described in detail^1,29^. Briefly, participants were included between 1 September 1987 and 31 January 2001, forming a surgery group (2010 patients, who all chose surgical treatment) and a matched control group (2037 patients) with identical inclusion and exclusion criteria (*Fig. 1*). The inclusion criteria were being aged 37–60 years and having a BMI of ≥34 kg/m^2^ for men and ≥38 kg/m^2^ for women, and all participants were eligible for surgery.

The exclusion criteria included an earlier operation for a gastric or duodenal ulcer, earlier MBS, a gastric ulcer during the past 6 months, ongoing malignancy, myocardial infarction during the past 6 months, active malignancy during the past 5 years, a bulimic eating pattern, drug or alcohol abuse (>0.75 l of 40% liquor per week or corresponding ethanol amount), psychiatric or cooperative problems contraindicating MBS, and other contraindicating conditions (such as continuous glucocorticoid or anti-phlogistic treatment).

Three of the patients originally included in the surgery group never underwent surgery and in the per-protocol analysis in the present study they were therefore included in the control group. Surgery patients underwent GBP, gastric banding, or vertical banded gastroplasty, and the type of surgery was determined by surgeons at the participating surgical departments. A matched control group was created using 18 matching variables (*Fig. 1*) according to the method of sequential treatment assignment^30^. Participants in the control group received conventional obesity care at their primary healthcare centre. The study began on the day of surgery for patients in the surgery group, as well as for their matched controls.

Baseline data on alcohol consumption were obtained from SOS questionnaires as previously described^10^. In brief, information on the total mean alcohol intake in g/day was calculated from the validated SOS dietary questionnaire^31^, which included questions on habitual intake of food and beverages during the previous 3 months.Participants who reported alcohol consumption >40 g/day for men (corresponding to approximately 3 standard drinks per day) or >20 g/day for women (corresponding to approximately 1.5 standard drinks per day) were classified as having medium- or high-risk alcohol consumption, as defined by the WHO^10,32^. A positive answer to the question ‘Do you think you have alcohol problems?’ was used to classify participants with self-reported alcohol problems. Although individuals with alcohol abuse were ineligible for the SOS study, some with high alcohol consumption, self-reported alcohol problems, or diagnosed AUD at baseline were included. At the time of study initiation in the late 1980s, alcohol-related surgical risks were not well recognized and eligibility was determined by the attending physician. Subsequent register linkage revealed some participants had undisclosed alcohol-related diagnoses at baseline.

The seven ethics review boards in Sweden (Lund, Gothenburg, Linköping, Karolinska Institute, Örebro, Uppsala, and Umeå) approved the study protocol. All participants provided written or oral informed consent. The study was registered at ClinicalTrials.gov (NCT01479452).

### Outcomes and follow-up

The primary endpoint of the SOS study was overall mortality^29^. In the original SOS study protocol from 1987 it was stated that negative effects of medical and surgical treatments of obesity should be reported. The present report examines both incidence of AUD diagnoses and alcohol-related mortality. Data on primary and secondary AUD diagnoses, recorded during inpatient or outpatient specialist care visits for any reason, were retrieved from the Swedish National Patient Register using ICD 9 and 10 codes (*Table S1*). To capture data on alcohol-related mortality during follow-up, information on all deaths was obtained by cross-checking the SOS database against the Swedish Population Register. Information on alcohol-related mortality was captured from the Swedish Cause of Death Register (death certificates), relevant case sheets, and autopsy reports. Alcohol-related deaths were defined as fatalities where alcohol was a major, though not necessarily the sole, contributing factor, as indicated by ICD codes linked to such a cause or explicitly noted in the underlying or contributory causes.

At the time of register linkage, the Swedish National Patient Register, which covers 99% of all hospital admissions and discharges^33^, contained complete information until the end of year 2022. The length of follow-up for this report was up to 35.1 years, with a median of 25.2 (i.q.r. 19.8 to 28.8) years.

### Statistical analysis

Mean(s.d.) values are used to describe baseline characteristics of the participants. Differences between group means were analysed using one-way ANOVA (continuous variables) or Fisher’s exact test (dichotomous variables). Time to first event (the first time a patient was diagnosed with an ICD code related to AUD or alcohol-related death) was calculated from the date of inclusion in the study. Those without an AUD diagnosis in the Swedish National Patient Register were treated as censored observations at the end of follow-up. Time to first event after inclusion was compared between the four treatment groups (GBP, VBG, gastric banding, and control) using Kaplan–Meier estimates of cumulative incidence rates (IRs). The proportional-hazards assumption was evaluated by assessing the interaction between treatment and the logarithm of time. A log rank test was used to analyse differences in cumulative incidence. A Cox proportional-hazards model or a competing-risks regression model suggested by Fine and Gray^34^ was used to evaluate time to an event, while adjusting for preselected risk factors for overconsumption of alcohol (sex and age, daily smoking, alcohol consumption and total calorie intake at baseline, and year of inclusion in the study). For the main analysis, a per-protocol approach was applied. All participants remained in their original study group until either MBS was performed in the usual care group or reoperations were performed that involved a change in surgical procedure or the restoration of normal anatomy in surgery patients. At that point, they were censored from the analysis. Data on surgeries during follow-up were obtained through linkage with the Swedish National Patient Register, as well as from standardized questionnaires completed by each patient at every follow-up visit.

A total of 36 paticipants with an AUD diagnosis before inclusion were excluded from the main analysis (*Fig. 1*). A sensitivity analysis also excluding 74 participants with high alcohol consumption and/or self-reported alcohol-related problems at baseline was conducted (*Fig. 1*). Additionally, to address potential confounding by baseline psychiatric co-morbidities, a separate sensitivity analysis was performed excluding 645 participants with a history of psychiatric care or psychotropic medication use (*Fig. 1*). In addition, a sensitivity analysis was conducted using time-varying covariates to evaluate whether undergoing GBP during follow-up was associated with AUD incidence and alcohol-related mortality. This analysis included participants who underwent GBP after study inclusion, either controls who later underwent GBP or VBG/gastric banding patients converted to GBP, analysed as a single group. In this analysis, participants who had undergone GBP at baseline were excluded. Time-at-risk IRs were calculated from the date of GBP surgery (*Fig. 1*).

For all analyses, *P* < 0.050 was considered statistically significant. Statistical analyses were carried out using the Stata statistical package, version 18.0 (StataCorp, College Station, TX, USA).

## Results

### Baseline characteristics of study participants

Within the per-protocol surgery group, 266 participants underwent GBP, 376 participants underwent gastric banding, and 1365 participants underwent VBG. At baseline, the patients in these three surgery groups were, on average, heavier (*P* < 0.001), younger (*P* < 0.001), and more frequently smokers (*P* = 0.001), compared with the participants in the control group (*Table 1*). There were no significant baseline differences in alcohol-related parameters between the control group and the three surgery groups, including mean alcohol consumption (*P* = 0.157), prevalence of at least medium-risk alcohol consumption as defined by the WHO (*P* = 0.141)^32^, self-reported alcohol problems (*P* = 0.792), and the proportion of participants reporting any alcohol consumption (*P* = 0.269).

**Table 1:** Baseline characteristics of surgically treated and control patients (per protocol)

	GBP	Gastric banding	VBG	Control	P*
Individuals, *n*	266	376	1365	2040	–
**Sex, *n***					0.893
Male	76	116	395	593	
Female	190	260	970	1447	
Age (years), mean(s.d.)	47.0(6.0)	47.6(6.0)	47.2(5.9)	48.7(6.2)	<0.001
Weight (kg), mean(s.d.)	125.1(19.2)	120.2(16.3)	120.3(16.1)	114.7(16.5)	<0.001
Height (m), mean(s.d.)	1.69(0.09)	1.70(0.09)	1.69(0.09)	1.69(0.09)	0.190
BMI (kg/m^2^), mean(s.d.)	43.8(5.2)	41.7(4.3)	42.3(4.3)	40.1(4.7)	<0.001
Daily smoking	62 (23.3)	105 (27.9)	352 (25.8)	426 (20.9)	0.001
History of psychiatric care or psychotropic medication	49 (18.4)	76 (20.2)	230 (16,0.8)	324 (15.9)	0.179
Alcohol consumption (g/day), mean(s.d.)	5.5(7.2)	5.9(7.8)	4.9(7.1)	5.3(8.1)	0.157
Any alcohol consumption	209 (78.6)	295 (78.5)	1016 (74.4)	1537 (75.4)	0.269
At least medium-risk alcohol consumption†	5 (1.9)	6 (1.6)	14 (1.0)	41 (2.0)	0.141
Self-reported alcohol problems†	0 (0.0)	2 (0.5)	8 (0.6)	11 (0.5)	0.792
Diagnosis in register before inclusion‡	3 (1.1)	2 (0.5)	15 (1.1)	16 (0.8)	0.639

Values are *n* (%) unless otherwise indicated. *One-way ANOVA (continuous variables) or Fisher’s exact test (dichotomous variables), comparing all four treatment types in the SOS intervention study. †Exclusion due to alcohol problems was the responsible doctor’s decision. A small number of patients with at least medium-risk alcohol consumption and/or self-reported problems were accepted. ‡Excluded from the main cumulative incidence analyses. GBP, gastric bypass; VBG, vertical banded gastroplasty.

### Incidence of AUD and weight trajectories during follow-up

**Fig. 2:**
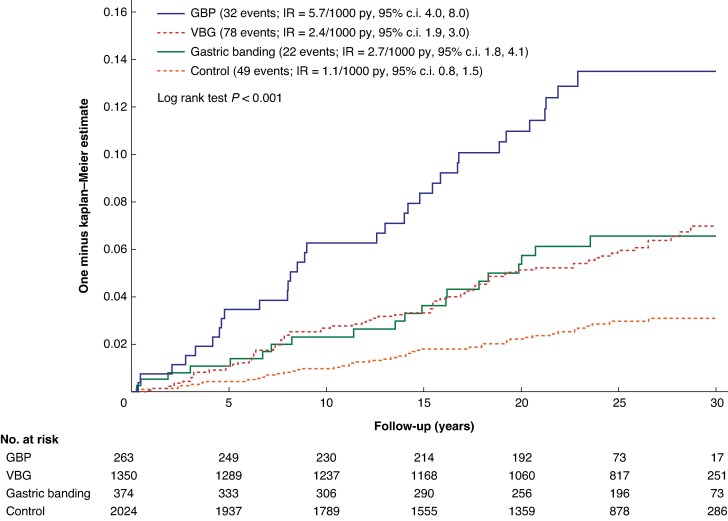
Unadjusted cumulative incidence of AUD diagnoses stratified by treatment type Follow-up in the figure is truncated at 30 years, but observations after this time point were included in the analyses. The median length of follow-up was 24.7 (i.q.r. 16.7 to 28.3) years for the control group, 26.3 (i.q.r. 18.7 to 29.7) years for the gastric banding group, 26.7 (i.q.r. 22.9 to 29.3) years for the VBG group, and 23.6 (i.q.r. 22.1 to 25.9) years for the GBP group. AUD, alcohol use disorder; GBP, gastric bypass; IR, incidence rate; py, person-years; VBG, vertical banded gastroplasty; i.q.r., interquartile range.

During follow-up, there were 181 participants with a documented AUD diagnosis in the Swedish National Patient Register (*Fig. 2*). The incidence of postoperative AUD differed significantly between treatment groups (log rank *P* < 0.001). Patients who underwent GBP exhibited the highest IRs of postoperative AUD with 5.7 (95% c.i. 4.0 to 8.0) events per 1000 person-years, compared with 1.1 (95% c.i. 0.8 to 1.5) events per 1000 person-years in the usual care group. In comparison, the IRs were 2.4 (95% c.i. 1.9 to 3.0) events per 1000 person-years after VBG and 2.7 (95% c.i. 1.8 to 4.1) events per 1000 person-years after gastric banding. IRs stratified by 5-year follow-up intervals are shown in *Table S2*. In multivariable analyses adjusted for preselected risk factors, both smoking and high alcohol intake at baseline were each independently linked with a higher risk of being diagnosed with AUD during follow-up (*Table 2*). Moreover, all surgical procedures were associated with a higher risk of AUD compared with usual care, with the highest risk observed after GBP (adjusted HR (HRadj) 5.07 (95% c.i. 3.11 to 8.25); *P* < 0.001). The corresponding HRadj values for VBG and gastric banding were 2.28 (95% c.i. 1.56 to 3.34) (*P* < 0.001) and 2.34 (95% c.i. 1.37 to 4.01) (*P* = 0.002) respectively (*Table 2*). There was no indication of violation of the proportional-hazards assumption (*P* = 0.950 for the test of interaction with time).

**Table 2:** Multivariable associations between baseline factors and risk of AUD and alcohol-related mortality

Variable	AUD* (181 events), HRadj (95% c.i.)	Alcohol-related mortality† (58 events), sub-HRadj (95% c.i.)
Sex (male *versus* female)	1.41 (0.96,2.06)	1.52 (0.78,2.93)
Age (per 5 years)	0.90 (0.79,1.01)	0.97 (0.81,1.17)
Smoking (yes *versus* no)	2.00 (1.47,2.72)	1.85 (1.07,3.20)
Alcohol g/day (per 10 g)	1.91 (1.66,2.20)	2.00 (1.61,2.49)
Total calorie intake kcal (per 500 kcal)	1.02 (0.96,1.09)	0.99 (0.87,1.12)
Inclusion year	1.01 (0.96,1.06)	0.99 (0.91,1.08)
**Treatment *versus* control (per protocol)**		
Control	Reference	Reference
Gastric banding	2.34 (1.37,4.01)	2.52 (0.89,7.15)
VBG	2.28 (1.56,3.34)	3.56 (1.79,7.08)
GBP	5.07 (3.11,8.25)	6.18 (2.48,15.40)

^*^Values are derived from a Cox proportional-hazards model. †Values are derived from a competing-risks regression model. AUD, alcohol use disorder; VBG, vertical banded gastroplasty; GBP, gastric bypass.

Moreover, a sensitivity analysis excluding 74 participants with high alcohol consumption and/or self-reported alcohol-related problems at baseline supported the findings of the main analysis (*Fig. S1*). Furthermore, to account for baseline psychiatric co-morbidities, a separate sensitivity analysis excluded 645 participants with a history of psychiatric care or psychotropic medication use. While results were consistent with the overall cohort, the incidence of AUD diagnoses during follow-up was lower in this subgroup (*Fig. S2*).

In an additional multivariable sensitivity analysis, time-varying covariates were included to specifically examine participants who underwent GBP during follow-up, that is controls who underwent GBP and VBG/gastric banding patients who were converted to GBP. In this analysis, participants who had undergone GBP at study inclusion were excluded. Overall, 624 participants underwent GBP during follow-up, of whom 173 were diagnosed with AUD. In this analysis, GBP surgery, treated as a time-dependent variable, remained a highly significant predictor of AUD compared with conventional obesity care (HRadj 4.68 (95% c.i. 3.01 to 7.29); *P* < 0.001). IRs of AUD were similar across the two GBP cohorts, with 5.7 (95% c.i. 4.0 to 8.0) events per 1000 person-years observed in patients who underwent GBP at baseline and 5.6 (95% c.i. 4.3 to 7.3) events per 1000 person-years observed in patients who underwent GBP during follow-up, that is controls who underwent GBP and VBG/gastric banding patients who were converted to GBP.

Ten-year weight trajectories stratified by AUD status for the control group and the three surgical groups are displayed in *Fig. S3*. In the VBG group, patients who developed AUD experienced significantly greater weight loss over the 10-year interval compared with those without AUD (*P* < 0.001). No significant differences in weight change were observed between patients who developed AUD and those without AUD in the other surgical groups or the control group.

### Alcohol-related mortality

**Fig. 3:**
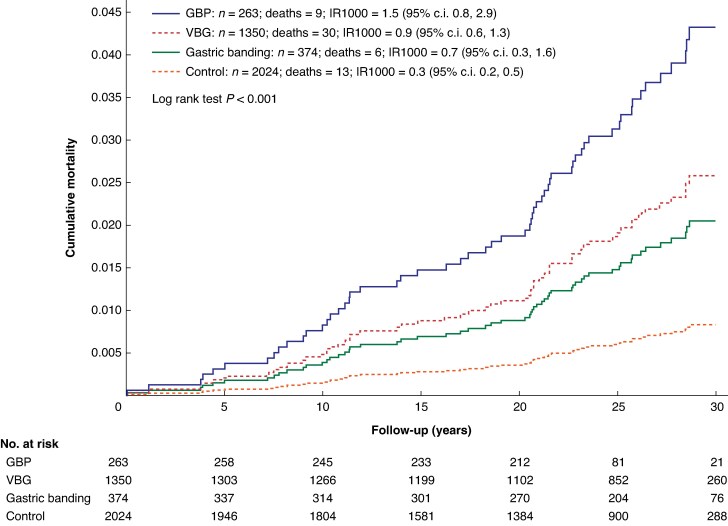
Unadjusted cumulative incidence of alcohol-related mortality stratified by treatment type Follow-up in the figure is truncated at 30 years, but observations after this time point were included in the analyses. GBP, gastric bypass; IR1000, incidence rate per 1000 person-years; VBG, vertical banded gastroplasty.

During follow-up, there were a total of 45 alcohol-related deaths in the surgery group and 13 in the control group and mortality rates were statistically significantly between treatment groups (log rank *P* < 0.001) (*Fig. 3*). Patients who underwent GBP exhibited the highest alcohol-related mortality rates with 1.5 (95% c.i. 0.8 to 2.9) deaths per 1000 person-years, compared with 0.3 (95% c.i. 0.2 to 0.5) deaths per 1000 person-years in the usual care group. After VBG and gastric banding, the mortality rates were 0.9 (95% c.i. 0.6 to 1.3) deaths per 1000 person-years and 0.7 (95% c.i. 0.3 to 1.6) deaths per 1000 person-years respectively.

In the multivariable analysis, smoking and high alcohol intake at baseline were independently associated with a higher risk of alcohol-related mortality during follow-up (*Table 2*). Furthermore, GBP and VBG were associated with a higher risk of alcohol-related mortality compared with controls (sub-HRadj 6.18 (95% c.i. 2.48 to 15.40) (*P* < 0.001) and sub-HRadj 3.56 (95% c.i. 1.79 to 7.08) (*P* < 0.001) respectively) (*Table 2*). Alcohol-related mortality was also higher in the gastric banding group compared with usual care, but the difference was not statistically significant (sub-HRadj 2.52 (95% c.i. 0.89 to 7.15); *P* = 0.082).

The association between GBP and alcohol-related mortality was further investigated through a sensitivity analysis employing a time-varying covariate approach. This analysis confirmed that, compared with usual care, GBP during follow-up was associated with an increased risk of alcohol-related mortality (sub-HRadj 4.42 (2.10 to 9.27); *P* < 0.001).

## Discussion

This study provides evidence that GBP is not only associated with an increased risk of alcohol-use disorder but also with a significantly elevated risk of alcohol-related mortality. Additionally, the risks of AUD and alcohol-related mortality were also observed to increase after VBG and gastric banding surgeries, although these increased risks were less pronounced.

Worldwide, approximately 5% of all deaths are attributed to alcohol consumption, with both acute (for example intoxications and accidents) and chronic (for example liver damage, heart failure, and cancer) aspects contributing to these high numbers^35^. There is a clear association between the level of alcohol consumption and alcohol-related mortality in the general population^36^. An association between MBS and AUD has been reported previously^7–10^, a finding corroborated by the present analysis. Building on this evidence, the present study also demonstrates a clear link between MBS and alcohol-related mortality, underscoring the seriousness of long-term alcohol-related complications after surgery. A previous study indicated that GBP patients have an increased risk of drug- and alcohol-related mortality compared with the general US population^37^. However, that study was limited by a small number of events and a relatively short follow-up interval of up to 7 years. The present findings extend this evidence by demonstrating a substantially increased risk of alcohol-related mortality after GBP in a prospective study with a matched control group and up to 35 years of follow-up. Notably, the robustness of this association was confirmed through sensitivity analyses. In addition, more modest increases in the risk of both AUD and alcohol-related mortality were observed among patients who underwent VBG or gastric banding, suggesting that these risks may not only be associated with GBP.

After early reports on altered alcohol metabolism^4–6^ and a potential increased risk of AUD after GBP^7–10^, a significant number of publications subsequently addressed this topic^27^. These studies have employed various designs and different outcome measures to investigate the possible association between MBS and AUD. For studies using diagnosis-based outcomes, such as retrospective register studies, the length of follow-up is a critical aspect of study design^27^. The development of addictive behaviours often occurs gradually and it may take a prolonged time for such behaviours to come to clinical attention and be formally diagnosed within the healthcare system. Furthermore, most MBS centres currently recommend abstaining from or reducing alcohol consumption after surgery. While these recommendations are likely followed closely immediately after the procedure, compliance may decrease over time. Consequently, studies with a short follow-up interval may fail to detect an association between MBS and AUD. Caution is therefore warranted when interpreting and publishing such findings, as they may underestimate long-term risks and lead to misleading conclusions regarding the impact of MBS on alcohol-related outcomes.

The increased risk of AUD and alcohol-related mortality observed after GBP is likely driven by a combination of increased alcohol sensitivity, altered alcohol metabolism, and enhanced reinforcing effects of alcohol after surgery^38^. However, a statistically significant increase in AUD was also observed after VBG and gastric banding. This indicates that other mechanisms, such as the ‘addiction transfer hypothesis’^39^, underlying psychological issues^40^, hormonal changes^41^, alterations in brain reward processing^41^, increased socialization in alcohol-related settings after weight loss, or the physiological effects of reduced body size, may also contribute to the association between MBS and AUD. All in all, further research is needed to clarify the underlying mechanisms, particularly those not specific to GBP. Moreover, recent progress in the pharmacological treatment options for obesity include glucagon-like peptide 1 receptor agonists (GLP-1RAs). Interestingly, GLP-1RAs not only promote weight loss but also appear to reduce cravings and the incidence of addictive behaviours, likely through their effects on the brain’s reward pathways^42^. This dual action suggests that GLP-1RAs may hold potential benefits for individuals with AUD or those at increased risk of developing it.

This study has limitations. First, the Swedish National Patient Register includes data from inpatient care and outpatient specialist care, but not from primary care or other outpatient clinics. As a result, the total incidence of AUD diagnoses may be underestimated, which may partly explain the low absolute IRs. However, the registers are known for their high coverage and data quality^33^, which is a strength of the study. However, missed outpatient AUD diagnoses would likely affect all study groups similarly, minimizing the risk of bias. Second, the study has no data on patients who underwent sleeve gastrectomy, as the SOS study only included surgical procedures that were commonly performed during the recruitment interval (1987–2001). Third, a randomized design was not approved by the ethics review boards at the time of the start of the SOS study. Treatment allocation was therefore the choice of the patient, which could result in bias not fully compensated by the matching procedure. In addition, the relatively small number of patients who had undergone GBP at baseline was partially addressed through time-varying covariate analyses. These analyses specifically included participants who underwent GBP during follow-up, that is controls who underwent GBP and VBG/gastric banding patients who were converted to GBP. Finally, the generalizability of the findings may also be limited by the predominantly Caucasian study population and the inclusion of participants that may not fulfil the current International Federation for the Surgery of Obesity and Metabolic Disorders (IFSO)/American Society of Metabolic and Bariatric Surgery (ASMBS) AUD exclusion criteria^43^ . Major strengths of the study include the large number of well-characterized participants, a controlled prospective design, and a median follow-up of 25 years.

The results of the present study strengthen and extend the previously described association between MBS and AUD. Moreover, the present study now demonstrates an increased risk of alcohol-related mortality after GBP, as well as after other MBS procedures. These results emphasize the importance of thorough pre-surgical screening and the education of both patients and healthcare professionals about potential post-surgical risks, while also underscoring the need for comprehensive post-surgical monitoring and tailored care to mitigate the risks of AUD and alcohol-related mortality.

## Supplementary Material

znaf211_Supplementary_Data

## Data Availability

Any requested information is subject to legal restrictions according to national legislation. Confidentiality regarding personal information in studies is regulated in the Public Access to Information and Secrecy Act (SFS 2009:400), OSL. A request to get access to public documents can be rejected or granted with reservations by the University of Gothenburg. If the University of Gothenburg refuses to disclose the documents the applicant is entitled to get a written decision that can be appealed to the administrative court of appeal.

## References

[1] Carlsson LMS, Sjöholm K, Jacobson P, Andersson-Assarsson JC, Svensson P-A, Taube M et al Life expectancy after bariatric surgery in the Swedish Obese Subjects study. N Engl J Med 2020;383:1535–1543 33053284 10.1056/NEJMoa2002449 PMC7580786

[2] Arterburn DE, Telem DA, Kushner RF, Courcoulas AP. Benefits and risks of bariatric surgery in adults: a review. JAMA 2020;324:879–887 32870301 10.1001/jama.2020.12567

[3] Thereaux J, Lesuffleur T, Czernichow S, Basdevant A, Msika S, Nocca D et al Long-term adverse events after sleeve gastrectomy or gastric bypass: a 7-year nationwide, observational, population-based, cohort study. Lancet Diabetes Endocrinol 2019;7:786–795 31383618 10.1016/S2213-8587(19)30191-3

[4] Hagedorn JC, Encarnacion B, Brat GA, Morton JM. Does gastric bypass alter alcohol metabolism? Surg Obes Relat Dis 2007;3:543–548; discussion 548 17903777 10.1016/j.soard.2007.07.003

[5] Klockhoff H, Naslund I, Jones AW. Faster absorption of ethanol and higher peak concentration in women after gastric bypass surgery. Br J Clin Pharmacol 2002;54:587–591 12492605 10.1046/j.1365-2125.2002.01698.x PMC1874483

[6] Woodard GA, Downey J, Hernandez-Boussard T, Morton JM. Impaired alcohol metabolism after gastric bypass surgery: a case-crossover trial. J Am Coll Surg 2011;212:209–214 21183366 10.1016/j.jamcollsurg.2010.09.020

[7] Conason A, Teixeira J, Hsu CH, Puma L, Knafo D, Geliebter A. Substance use following bariatric weight loss surgery. JAMA Surg 2013;148:145–150 23560285 10.1001/2013.jamasurg.265

[8] King WC, Chen J-Y, Mitchell JE, Kalarchian MA, Steffen KJ, Engel SG et al Prevalence of alcohol use disorders before and after bariatric surgery. JAMA 2012;307:2516–2525 22710289 10.1001/jama.2012.6147 PMC3682834

[9] Östlund MP, Backman O, Marsk R, Stockeld D, Lagergren J, Rasmussen F et al Increased admission for alcohol dependence after gastric bypass surgery compared with restrictive bariatric surgery. JAMA Surg 2013;148:374–377 23716012 10.1001/jamasurg.2013.700

[10] Svensson P, Anveden Å, Romeo S, Peltonen M, Ahlin S, Burza MA et al Alcohol consumption and alcohol problems after bariatric surgery in the Swedish Obese Subjects study. Obesity (Silver Spring) 2013;21:2444–2451 23520203 10.1002/oby.20397

[11] Parikh M, Johnson JM, Ballem N; American Society for Metabolic and Bariatric Surgery Clinical Issues Committee. ASMBS position statement on alcohol use before and after bariatric surgery. Surg Obes Relat Dis. 2016;12:225–230 26968500 10.1016/j.soard.2015.10.085

[12] Backman O, Stockeld D, Rasmussen F, Naslund E, Marsk R. Alcohol and substance abuse, depression and suicide attempts after Roux-en-Y gastric bypass surgery. Br J Surg 2016;103:1336–1342 27467694 10.1002/bjs.10258

[13] King WC, Chen J-Y, Courcoulas AP, Dakin GF, Engel SG, Flum DR et al Alcohol and other substance use after bariatric surgery: prospective evidence from a U.S. multicenter cohort study. Surg Obes Relat Dis 2017;13:1392–1402 28528115 10.1016/j.soard.2017.03.021 PMC5568472

[14] Maciejewski ML, Smith VA, Berkowitz TSZ, Arterburn DE, Mitchell JE, Olsen MK et al Association of bariatric surgical procedures with changes in unhealthy alcohol use among US veterans. JAMA Netw Open 2020;3:e2028117 33346846 10.1001/jamanetworkopen.2020.28117 PMC7753905

[15] Bramming M, Becker U, Jorgensen MB, Neermark S, Bisgaard T, Tolstrup JS. Bariatric surgery and risk of alcohol use disorder: a register-based cohort study. Int J Epidemiol 2020;49:1826–1835 10.1093/ije/dyaa147 33085738

[16] Mellinger JL, Shedden K, Winder GS, Fernandez AC, Lee BP, Waljee J et al Bariatric surgery and the risk of alcohol-related cirrhosis and alcohol misuse. Liver Int 2021;41:1012–1019 33529460 10.1111/liv.14805 PMC8204517

[17] Mahmud N, Panchal S, Abu-Gazala S, Serper M, Lewis JD, Kaplan DE. Association between bariatric surgery and alcohol use-related hospitalization and all-cause mortality in a Veterans Affairs cohort. JAMA Surg 2023;158:162–171 36515960 10.1001/jamasurg.2022.6410 PMC9856780

[18] Kovacs Z, Valentin JB, Nielsen RE. Risk of psychiatric disorders, self-harm behaviour and service use associated with bariatric surgery. Acta Psychiatr Scand 2017;135:149–158 27864830 10.1111/acps.12669

[19] Svensson CJ, Giang KW, Wallert J, Ruck C, Lundberg CE. Psychiatric co-morbidity and substance abuse after gastric bypass surgery. Br J Surg 2023;110:1618–1622 37314045 10.1093/bjs/znad179 PMC10638527

[20] Butt M, Eisler RA, Hu A, Rogers AM, Rigby A. Incidence of substance use disorder following bariatric surgery: a retrospective cohort study. Obes Surg 2023;33:890–896 36477697 10.1007/s11695-022-06400-6

[21] Lent MR, Hayes SM, Wood GC, Napolitano MA, Argyropoulos G, Gerhard GS et al Smoking and alcohol use in gastric bypass patients. Eat Behav 2013;14:460–463 24183136 10.1016/j.eatbeh.2013.08.008 PMC3817413

[22] Alfonsson S, Sundbom M, Ghaderi A. Is age a better predictor of weight loss one year after gastric bypass than symptoms of disordered eating, depression, adult ADHD and alcohol consumption? Eat Behav 2014;15:644–647 25260133 10.1016/j.eatbeh.2014.08.024

[23] de Araujo Burgos MGP, Cabral PC, Maio R, Oliveira BMPM, Dias MSO, de Figueiredo Melim DB et al Prevalence of alcohol abuse before and after bariatric surgery associated with nutritional and lifestyle factors: a study involving a Portuguese population. Obes Surg 2015;25:1716–1722 25691351 10.1007/s11695-015-1609-7

[24] Hilgendorf W, Butler A, Timsina L, Choi J, Banerjee A, Selzer D et al A behavioral rating system predicts weight loss and quality of life after bariatric surgery. Surg Obes Relat Dis 2018;14:1167–1172 29853194 10.1016/j.soard.2018.04.012

[25] Azam H, Shahrestani S, Phan K. Alcohol use disorders before and after bariatric surgery: a systematic review and meta-analysis. Ann Transl Med 2018;6:148 29862237 10.21037/atm.2018.03.16 PMC5952017

[26] Capelo Vides M, Campello de Oliveira M, Lassi DLS, Malbergier A, Florio L, de Azevedo-Marques Périco C et al Bariatric surgery and its influence on alcohol consumption: differences before and after surgery - a systematic review and meta-analysis. Int Rev Psychiatr 2023;35:367–376 10.1080/09540261.2023.2223317 38299644

[27] Kenkre JS, Gesell S, Keller A, Milani RM, Scholtz S, Barley EA. Alcohol misuse post metabolic and bariatric surgery: a systematic review of longer-term studies with focus on new onset alcohol use disorder and differences between surgery types. Curr Obes Rep 2024;13:596–616 38850501 10.1007/s13679-024-00577-w PMC11306568

[28] Svensson P, Peltonen M, Andersson-Assarsson JC, Ahlin S, Brembeck P, Engström M et al Non-alcohol substance use disorder after bariatric surgery in the prospective, controlled Swedish Obese Subjects study. Obesity (Silver Spring) 2023;31:2171–2177 37475690 10.1002/oby.23800

[29] Sjöström L, Narbro K, Sjöström CD, Karason K, Larsson B, Wedel H et al Effects of bariatric surgery on mortality in Swedish obese subjects. N Engl J Med 2007;357:741–752 17715408 10.1056/NEJMoa066254

[30] Pocock SJ, Simon R. Sequential treatment assignment with balancing for prognostic factors in the controlled clinical trial. Biometrics 1975;31:103–115 1100130

[31] Lindroos AK, Lissner L, Sjostrom L. Validity and reproducibility of a self-administered dietary questionnaire in obese and non-obese subjects. Eur J Clin Nutr 1993;47:461–481 8404782

[32] WHO – Department of Mental Health and Substance Dependence . *International Guide for Monitoring Alcohol Consumption and Related Harm*. 2000. https://www.who.int/publications/i/item/international-guide-for-monitoring-alcohol-consumption-and-related-harm (accessed 29 June 2024)

[33] Ludvigsson JF, Andersson E, Ekbom A, Feychting M, Kim J-L, Reuterwall C et al External review and validation of the Swedish National Inpatient Register. BMC Public Health 2011;11:450 21658213 10.1186/1471-2458-11-450 PMC3142234

[34] Fine JP, Gray RJ. A proportional hazards model for the subdistribution of a competing risk. J Am Stat Assoc 1999;94:496–509

[35] WHO – Department of Mental Health and Substance Dependence . *Global Status Report on Alcohol and Health and Treatment of Substance Use Disorders*. 2024. https://www.who.int/publications/i/item/9789240096745 (accessed 29 June 2024)

[36] Carr T, Kilian C, Llamosas-Falcón L, Zhu Y, Lasserre AM, Puka K et al The risk relationships between alcohol consumption, alcohol use disorder and alcohol use disorder mortality: a systematic review and meta-analysis. Addiction 2024;119:1174–1187 38450868 10.1111/add.16456 PMC11156554

[37] White GE, Courcoulas AP, King WC. Drug- and alcohol-related mortality risk after bariatric surgery: evidence from a 7-year prospective multicenter cohort study. Surg Obes Relat Dis 2019;15:1160–1169 31182414 10.1016/j.soard.2019.04.007 PMC13011885

[38] Engel SG, Schaefer LM, Kerver GA, Leone LM, Smith G, Mitchell JE et al The rewarding effects of alcohol after bariatric surgery: do they change and are they associated with pharmacokinetic changes? Surg Obes Relat Dis 2022;18:190–195 34583891 10.1016/j.soard.2021.08.011 PMC8792168

[39] Yoder R, MacNeela P, Conway R, Heary C. How do individuals develop alcohol use disorder after bariatric surgery? A grounded theory exploration. Obes Surg 2018;28:717–724 29032488 10.1007/s11695-017-2936-7

[40] Castillo-Carniglia A, Keyes KM, Hasin DS, Cerda M. Psychiatric comorbidities in alcohol use disorder. Lancet Psychiatry 2019;6:1068–1080 31630984 10.1016/S2215-0366(19)30222-6 PMC7006178

[41] Blackburn AN, Hajnal A, Leggio L. The gut in the brain: the effects of bariatric surgery on alcohol consumption. Addict Biol 2017;22:1540–1553 27578259 10.1111/adb.12436 PMC5332539

[42] Jerlhag E . GLP-1 receptor agonists: promising therapeutic targets for alcohol use disorder. Endocrinology 2025;166:bqaf028 39980336 10.1210/endocr/bqaf028 PMC11879929

[43] Eisenberg D, Shikora SA, Aarts E, Aminian A, Angrisani L, Cohen RV et al 2022 American Society of Metabolic and Bariatric Surgery (ASMBS) and International Federation for the Surgery of Obesity and Metabolic Disorders (IFSO) indications for metabolic and bariatric surgery. Obes Surg 2023;33:3–14 36336720 10.1007/s11695-022-06332-1 PMC9834364

