# Supplementary material for: Alcohol use disorder and alcohol-related mortality after metabolic bariatric surgery: prospective controlled cohort study
Source: Br J Surg. 2025 Oct 9;112(10):znaf211. doi: 10.1093/bjs/znaf211 (PMC12509842; doi:10.1093/bjs/znaf211)
Supplement: znaf211_Supplementary_Data [file znaf211_supplementary_data.docx]

**Title:** Alcohol use disorder and alcohol-related mortality after metabolic bariatric surgery: A prospective controlled cohort study

**Authors:** Kajsa Sjöholm^1^, Markku Peltonen^2^, Peter Jacobson^1^, Johanna C Andersson-Assarsson^1^, Sofie Ahlin^1, 3^, Lucas Adméus^1^, Ida Arnetorp^1^, My Engström^4, 5^, Magdalena Taube^1^, Lena MS Carlsson^1^, Per-Arne Svensson^1, 5^

^1^Department of Molecular and Clinical Medicine; Institute of Medicine, Sahlgrenska Academy at University of Gothenburg, Gothenburg, Sweden.

^2^Finnish Institute for Health and Welfare, Helsinki, Finland

^3^Region Västra Götaland, NU hospital group, Department of Clinical Physiology, Trollhättan, Sweden

^4^Department of Surgery, Region Västra Götaland, Sahlgrenska University Hospital, Gothenburg, Sweden

^5^Institute of Health and Care Sciences, Sahlgrenska Academy at University of Gothenburg, Gothenburg, Sweden.

**Corresponding author:** Professor Per-Arne Svensson, Box 457, SE-405 30 Gothenburg, Sweden. E-mail: [per-arne.svensson@gu.se](mailto:per-arne.svensson@gu.se). ORCID ID, [0000-0002-6731-806X](https://orcid.org/0000-0002-6731-806X);

**Supplementary Materials Index**

| **Supplementary Figures and Tables** |  |
| --- | --- |
| **Figure S1.** Sensitivity analysis of cumulative incidence of alcohol use disorder diagnoses, excluding n=74 participants with medium-risk alcohol consumption and/or self-reported alcohol problems at study inclusion. | *Page 3* |
| **Figure S2.** Sensitivity analysis of cumulative incidence of alcohol use disorder diagnoses excluding n=645 participants with a history of psychiatric care or psychotropic medication use | *Page 4* |
| **Figure S3.** BMI development by intervention and AUD over 10 years. | *Page 5* |
| **Table S1.** International Classification of Disease (ICD)-9 and -10 codes | *Page 6* |
| **Table S2** Alcohol use disorder incidence rates, stratified by 5-year follow-up periods. | *Page 7* |
|  |  |

**Figure S1.** Sensitivity analysis of cumulative incidence of alcohol use disorder diagnoses, excluding n=74 participants with medium-risk alcohol consumption and/or self-reported alcohol problems at study inclusion. GBP, Gastric Bypass; VBG, Vertical Banded Gastroplasty.

**Figure S2.** Sensitivity analysis of cumulative incidence of alcohol use disorder diagnoses excluding n=645 participants with a history of psychiatric care or psychotropic medication use. GBP, Gastric Bypass; VBG, Vertical Banded Gastroplasty.

**Figure S3.** BMI development in patients treated with GBP (a), banding (b), VBG (c), and usual care controls (d), stratified by alcohol use disorder diagnoses during follow-up. Figures contain estimated means from a mixed model with adjustment for sex and age. GBP, Gastric Bypass; VBG, Vertical Banded Gastroplasty.

**Table S1.** International Classification of Disease (ICD)-9 and -10 codes used for assessment of alcohol use disorder diagnosis and alcohol-related mortality

| **Description** | **ICD-9** | **ICD-10** |
| --- | --- | --- |
| Mental and behavioral disorders due to alcohol use | 291 | F10 |
| Alcohol dependence syndrome | 303 | F10.2 |
| Alcohol abuse | 305.0 | F10 |
| Alcoholic polyneuropathy | 357.5 | G62.1 |
| Alcoholic cardiomyopathy | 425.5 | I42.6 |
| Alcoholic myopathy | - | G72.1 |
| Alcoholic gastritis | 535.3 | K29.2 |
| Alcoholic liver disease | 571.0-3 | K70.0-4, K70.9 |
| Special screening for alcoholism | V79.1 | Z13.3 |
| Alcohol induced pancreatitis | - | K85.2, K86.0 |
| Degeneration of nervous system due to alcohol | - | G31.2 |
| Alcohol abuse counseling and surveillance | - | Z71.4 |
| Problems related to lifestyle: alcohol use | - | Z72.1 |

**Table S2.** Alcohol use disorder incidence rates, stratified by 5-year follow-up periods.

| **Incidence rates/1000 p-y** | | | | |
| --- | --- | --- | --- | --- |
| Time interval, years | Control | Banding | VBG | GBP |
| <=5 | 0.9 | 2.3 | 2.1 | 7.0 |
| 5-10 | 1.1 | 2.5 | 3.3 | 5.8 |
| 10-15 | 1.7 | 2.7 | 1.3 | 4.4 |
| 15-20 | 0.8 | 3.6 | 3.8 | 5.8 |
| 20-25 | 1.5 | 2.6 | 1.7 | 6.9 |
| >25 | 0.3 | 2.3 | 1.9 | (n.a.) |

GBP, Gastric Bypass; VBG, Vertical Banded Gastroplasty; p-y, person-years.
